# Cancer Chemopreventive Properties of Glutelin Hydrolysate from Riceberry Bran Residues Against the Early Stage of Liver and Colon Carcinogenesis Induced by Chemicals in Rats

**DOI:** 10.3390/cancers17162666

**Published:** 2025-08-15

**Authors:** Aroonrat Pharapirom, Sirinya Taya, Arpamas Vachiraarunwong, Warunyoo Phannasorn, Chonikarn Singai, Rawiwan Wongpoomchai, Jetsada Ruangsuriya

**Affiliations:** 1 Department of Biochemistry, Faculty of Medicine, Chiang Mai University, Chiang Mai 50200, Thailand; aroonrat_p@cmu.ac.th (A.P.); arpamas.vachi@omu.ac.jp (A.V.); warunyoo.p@cmu.ac.th (W.P.); chonikarn.s@gmail.com (C.S.); rawiwan.wong@cmu.ac.th (R.W.); 2 Functional Food Research Unit, Multidisciplinary Research Institute, Chiang Mai University, Chiang Mai 50200, Thailand; 3 Department of Environmental Risk Assessment, Graduate School of Medicine, Osaka Metropolitan University, Osaka 545-8585, Japan; 4 Center of Multidisciplinary Technology for Advanced Medicine (CMUTEAM), Faculty of Medicine, Chiang Mai University, Chiang Mai 50200, Thailand

**Keywords:** riceberry bran protein, glutelin, glutelin hydrolysate, cancer chemoprevention, glutathione *S*-transferase placental form-positive foci, aberrant crypt foci

## Abstract

**Background**: Rice bran proteins and their hydrolysates exhibit anticancer activity. Our previous study demonstrated that Riceberry glutelin and its hydrolysates possessed potent in vitro antioxidant and antimutagenic properties. However, their cancer chemopreventive effects in animals remain unclear. **Methods**: This study investigated chemopreventive mechanisms in diethylnitrosamine (DEN)- and 1,2-dimethylhydrazine (DMH)-induced preneoplastic lesions, including glutathione *S*-transferase placental form (GST-P)-positive foci in the liver and aberrant crypt foci (ACF) in the colon of rats. Rats received GTL, GTLH, and total protein hydrolysate (TPH) at 500 mg/kg body weight, five days per week for ten weeks. **Results**: GTLH significantly reduced GST-P-positive foci in the liver and ACF in the colon, while GTL decreased GST-P-positive foci only in the liver. However, TPH did not affect preneoplastic lesions in both the liver and the colon. GTLH suppressed cell proliferation by reducing proliferating cell nuclear antigen (PCNA)-positive cells and promoted apoptosis, as indicated by an increase in terminal deoxynucleotidyl transferase dUTP nick-end labeling (TUNEL)-positive cells in both organs. GTL also decreased PCNA-positive cells in the liver and colon. Moreover, GTLH significantly upregulated *BAX* and *CASP3* in the liver, while only *BAX* in the colon was observed. **Conclusions**: This study highlighted the cancer chemopreventive potential of Riceberry GTLH with its underlying mechanism to reduce the number of preneoplastic lesions in the liver and colon through cell proliferation inhibition and apoptosis induction. These findings suggested that this protein hydrolysate might be used as a functional food ingredient or dietary supplement for cancer prevention.

## Simple Summary

Cancer chemoprevention has become a promising alternative against cancer. Various biological activities, such as antioxidants, anti-inflammation, antimutagenicity, antiproliferation, and apoptotic induction, are the properties of cancer chemopreventive agents. We proposed in this study that Riceberry glutelin hydrolysate (GTLH), not total protein hydrolysate, had cancer chemopreventive properties against liver and colon cancers by reduction of preneoplastic lesion incidence in the liver and the colon of rats exposed to carcinogens with possible mechanisms involving the inhibition of cell proliferation, activation of apoptotic gene expression, and promotion of cancer cell apoptosis.

## 1. Introduction

Healthy diet consumption has become a strategic trend of human health promotion and disease prevention, especially for non-communicable diseases (NCDs) such as cancer. Specifically, cancer chemoprevention is of interest for preventing the emergence of cancer rather than the treatments. Cancer chemoprevention can affect multiple stages of carcinogenesis, including initiation, promotion, and progression, by delaying, reversing, and suppressing cancer cell development depending on the underlying mechanisms of each substance [1,2]. Furthermore, cancer chemopreventive agents can be categorized into two types, which are blocking agents and suppressing agents. The bioactive compounds inhibiting the initiation stage are known as blocking agents, while inhibiting the promotion and/or the progression stage are recognized as suppressing agents [2].

Cancer is the second leading cause of death worldwide. In 2022, the top three cancer types with the highest mortality rates were lung, colorectal, and liver cancers [3]. These cancers are related to the modern civilization lifestyle because of frequent exposure to various mutagens and/or carcinogens contaminating the environment, especially in the air and diets [4]. For example, 90% of lung cancer cases have been reported to be associated with cigarette smoke exposure [5]. Regular consumption of processed food is a well-known risk factor for colorectal cancer [6]. In addition, many hepatotoxins, such as aflatoxin, vinyl chloride, arsenic, and cadmium, that can contaminate dietary products, can lead to chronic liver disease and increase the risk of liver cancer [7]. The cancer risk factors associated with diets are difficult to avoid due to contamination, and consumption hardly causes acute symptoms that contribute to carcinogenesis. Nevertheless, the incidences of both colon and liver cancers have high mortality rates, and they have been predicted to rise in the near future [8]. Even worse, liver and colon cancers are often diagnosed at an advanced stage, in which the treatment options are limited [9,10]. Therefore, the study of cancer prevention becomes an intriguing field among scientists to provide evidence for the prevention of carcinogenesis, especially for liver and colon cancers.

In the past two decades, numerous nutrients and dietary constituents from plants and animals have been recognized for their effective anticancer properties, particularly proteins [11]. Proteins are essential nutrients for various physiological functions in the human body [12]. Among these, bioactive peptides, conserved sequences within proteins, have gained attention for their biological activities. Peptides consisting of 2–20 amino acids have demonstrated antioxidant, anti-inflammatory, anti-cholesterolemic, antiproliferative, apoptotic, and anticancer properties [13,14,15,16]. Additionally, peptides exhibit remarkable attributes such as specific targeting of cancer cells and excellent cell-penetrating abilities [17]. Several studies highlighted the chemopreventive potential of peptides derived from various dietary sources. For instance, peptides from common beans (*Phaseolus vulgaris* L.), which were used as a traditional diet and normally consumed in Mexico, including GLTSK, LSGNK, GEGSGA, MPACGSS, and MTEEY, inhibited cell proliferation in human colon cancer cell lines, namely RKO and HCT-116, by downregulation of glutathione redox-related enzymes in the RKO cell line and upregulation of NRF-2 related antioxidant enzymes [18]. Similarly, mung bean hydrolysate, which contained various tripeptides such as VEG, PQG, LAF, and EGA, had anticancer effects against human hepatocellular carcinoma cells, namely the HepG2 cell line, by inducing cell apoptosis via cell cycle arrest in the S phase and the G0/G1 phases at the low and high doses, respectively. Unlike 5-FU treatment, the tripeptides suppressed tumor cell proliferation in mice without affecting the changes in body weight [19]. Lunasin, a 43-amino-acid peptide found in soybeans, had antiproliferative activity in breast cancer cells by modulating inflammatory mediators, aromatase, and estrogen receptors [20]. In addition, proteins from edible grains such as rice, wheat, rye, oats, barley, millet, and maize also exhibited chemopreventive properties due to their antioxidant, anti-inflammatory, antiproliferative, and pro-apoptotic activities [21,22,23,24]. For example, peptides obtained from Alcalase-hydrolyzed maize albumin were shown to induce apoptosis in human hepatocellular carcinoma HepG2 cell lines, whereas quinoa-derived peptides suppressed cancer cell proliferation by inhibiting an enzyme regulating gene expression, namely histone deacetylase 1 (HDAC1), downregulating many inflammatory genes expression, and triggering apoptosis in a human colorectal adenocarcinoma cell line called Caco-2 [25,26]. These results collectively underscore the potential of dietary proteins and their peptides as promising candidates for cancer prevention. Rice bran is a by-product of rice milling. However, it still contains high-value proteins that can be separated by their solubility, including water-soluble albumin, salt-soluble globulin, alcohol-soluble prolamin, and alkaline-soluble glutelin. Glutelin (GTL) is a major protein in rice bran and has significant biological activities, including antioxidant, anti-inflammatory, and anticancer properties [27,28]. However, several studies investigated its biological activities in in vitro assays. Moreover, GTL hydrolysate (GTLH) contained pyroglutamyl peptides that increased bioactivities when compared with GTL. It presented bioactive effects such as antioxidant, anti-inflammation, and anticancer higher than GTL [29,30]. Similarly, our recently published findings showed that GTLH from Riceberry bran residues had antimutagenic activity using the Ames test [31]. However, the data on the cancer chemopreventive effect of Riceberry GTL and hydrolysates are scarce to understand its cancer chemoprevention properties, especially in animal models.

Preparation of GTL required a few steps of extraction, including alkaline extraction and pH precipitation [31]. To simplify the extraction of GTL for industrial applications, it was wondered if the GTL-containing portion, along with other proteins, which was known as total protein (TP), could have similar chemopreventive effects to GTL and/or GTLH. Therefore, this study aims to investigate the cancer chemopreventive effects of GTL, GTLH, and total protein hydrolysate (TPH) on diethylnitrosamine (DEN)- and 3,3′-diaminobenzidine tetrahydrochloride (DMH)-induced early stage of liver and colon carcinogenesis in rats, along with their potential preventive mechanisms.

## 2. Materials and Methods

### 2.1. Chemicals

Riceberry bran residues were kindly provided by the Kerk rice mill (Chiang Rai, Thailand). Diethylnitrosamine (DEN), 3,3′-diaminobenzidine tetrahydrochloride (DMH), and Flavourzyme^®^ were purchased from Sigma Aldrich (St. Louis, MO, USA). The ApopTag Peroxidase in situ kit, Alcalase^®^, and methylene blue were obtained from Merck (Darmstadt, Germany). 1,2-dimethylhydrazine dihydrochloride was purchased from Tokyo Chemical Industry Co., Ltd. (Tokyo, Japan). The rabbit polyclonal anti-rat GST-P and the mouse monoclonal anti-rat PCNA were purchased from MBL (Nagoya, Japan) and Biolegend (San Diego, CA, USA), respectively. An avidin–biotin–horseradish peroxidase complex (ABC) kit and a Vectastain Elite ABC kit (Universal) were purchased from Vector Laboratories Inc. (Burlingame, CA, USA). All chemicals were analytical grade.

### 2.2. Preparations of Proteins and Their Hydrolysates

**Figure 1:**
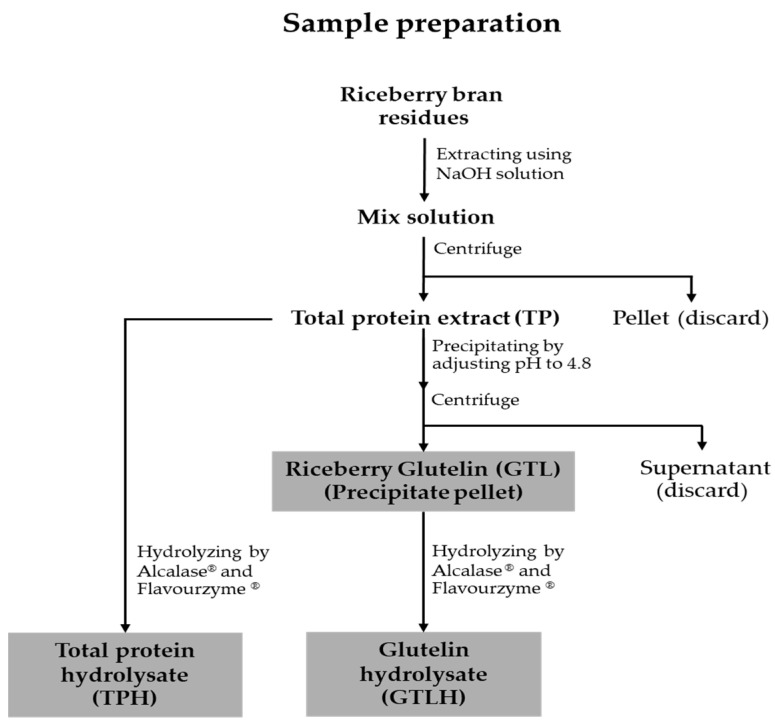
Schematic preparations of proteins and their hydrolysates.

Proteins and their hydrolysates were prepared according to the previously published method presented in Figure 1 [31]. The rice bran residues were soaked in 0.1 M NaOH for 4 h, followed by centrifugation at 6000× *g* for 15 min. The supernatant was adjusted to a pH of 4.8 to precipitate GTL. Additionally, the extract obtained without the precipitation process was classified as total protein (TP). All proteins were dried with a lyophilizer (FreeZone 2.5 L, Labconco, Kansas City, MO, USA) and stored at −20 °C for further hydrolysis and studies.

To prepare protein hydrolysates, two commercial enzymes, including 5% Alcalase^®^ and 2% Flavourzyme^®^, were used in sequential digestion due to the fact that using both enzymes increases protein hydrolysis efficiency and produces a wide range of peptides [29]. Alcalase, an endopeptidase, specifically cleaves peptide bonds at the carbonyl group of glutamic acid (Glu), methionine (Met), leucine (Leu), tyrosine (Tyr), lysine (Lys), and glutamine (Gln) [32]. In contrast, Flavourzyme, an exopeptidase, had no amino acid specificity [33]. In brief, 5 g of a protein sample was mixed with 25 mL of phosphate buffer saline (PBS) at pH 7.4. Then, the mixed solution was adjusted to pH 8.0, Alcalase^®^ was added at 5% (*v*/*w*), and the mixture was incubated at 50 °C for 4 h. After incubation, the mixture was adjusted to pH 7.0, and Flavourzyme^®^ was added at 2% (*v*/*w*). Again, the mixture was incubated at 50 °C for 4 h prior to inactivation in a water bath at 95 °C for 10 min. The supernatant was obtained by centrifugation at 6000× *g* for 10 min at 4 °C and mixed with 10% (*w*/*v*) maltodextrin for spray drying. GTLH and TPH were kept at −20 °C for further studies [29].

### 2.3. Animals

The cancer chemopreventive effects of GTL, GTLH, and TPH on liver and colon carcinogenesis induced by DEN and DMH were studied in Male Wistar rats aged three weeks (weight 80–100 g), which were purchased from Nomura Siam International Co., Ltd., Bangkok, Thailand. These rats were housed in a room maintained at 25 °C with 50% relative humidity and a 12 h light/dark cycle. The animal experimental protocol was approved by the Animal Ethics Committee of the Faculty of Medicine, Chiang Mai University, Thailand (Ethic number: 37/2562).

**Figure 2:**
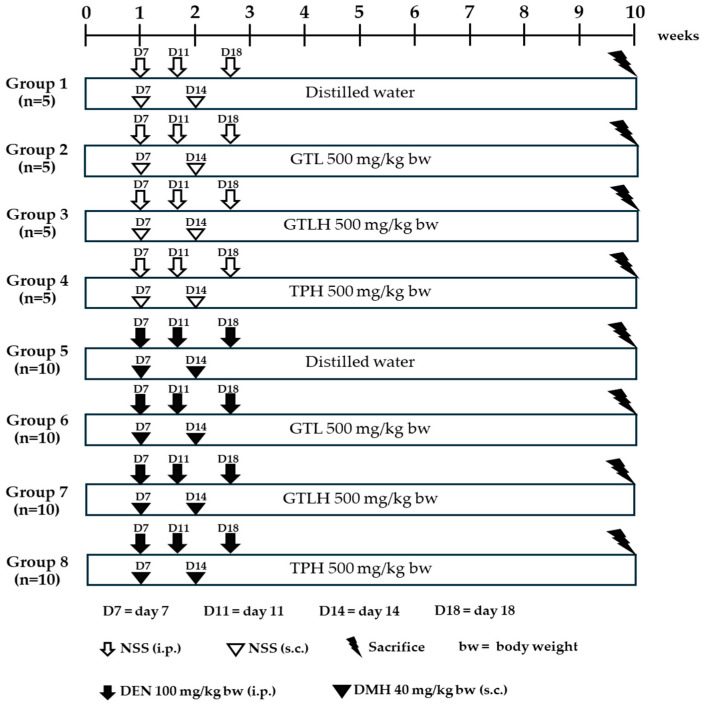
Animal experimental design for cancer chemoprevention of GTL, GTLH, and TPH in carcinogen-induced preneoplastic lesions in the liver and colon. NSS: normal saline solution; DEN: diethylnitrosamine; DMH: 1,2-dimethylhydrazine; GTL: glutelin; GTLH: GLT hydrolysate; TPH: total protein hydrolysate; i.p.: intraperitoneal injection; s.c.: subcutaneous injection.

Rats were randomly divided into 8 groups as illustrated in Figure 2. Groups 1–4 were normal saline solution (NSS)-treated rats, while groups 5–8 were carcinogen-treated rats. In order to study carcinogenic effects, groups 2–4 received 500 mg/kg bw GLT, GLTH, or TPH for 10 weeks, respectively. Similarly, to investigate the cancer chemopreventive effects, groups 6–8 also received 500 mg/kg bw GLT, GLTH, or TPH for 10 weeks, respectively. The water and the diet intakes were recorded throughout the experiment. Groups 5–8 were intraperitoneally injected with 100 mg/kg bw of DEN on days 7, 11, and 18 to initiate hepatocarcinogenesis, and subcutaneously injected with 40 mg/kg bw of DMH on days 7 and 14 to induce colon carcinogenesis, while groups 1–4 were injected with NSS. At the end of the experiment, all rats were euthanized with isoflurane. The internal organs, including the liver, kidneys, and spleen, were collected and weighed. The liver was cut apart and divided into two portions. One was flash-frozen and stored at −80 °C for molecular mechanism analyses, whereas another was fixed in 10% formaldehyde in PBS for immunohistochemistry. For colon tissue collection, rats in each group had their colons fixed in 10% formaldehyde. A 1 cm section of the formaldehyde-fixed colon was collected and embedded in paraffin for immunohistochemical analyses. The remaining fixed colons were rinsed with 0.9% normal saline solution, cut longitudinally into segments, and stained with methylene blue. For molecular mechanism analyses, mucosal cells were collected from the colon and stored at −80 °C.

### 2.4. Evaluation of Preneoplastic Lesions in the Liver and Colon

#### 2.4.1. Measurement of Glutathione *S*-Transferase Placental Form-Positive Foci in Liver Tissue by Immunohistochemistry

The immunohistochemistry of glutathione *S*-transferase placental form (GST-P)-positive foci in the liver was determined by the method of Puatanachokchai et al. [34]. Liver sections with a thickness of 4 µm were deparaffinized and rehydrated. Samples were treated with 3% hydrogen peroxide (H_2_O_2_) to block endogenous peroxidase activity. Subsequently, 1% skim milk solution was applied to prevent non-specific binding. The sections were then incubated with rabbit polyclonal anti-rat GST-P antibody (dilution 1:1000) and with anti-rabbit IgG biotinylated antibody, subsequently. The color was developed with DAB, and hematoxylin was used for counterstaining. The area and the number of GST-P-positive foci were measured using the (LAS) Interactive Measurement program (Leica Microsystems, Wetzlar, Germany).

#### 2.4.2. Measurement of Aberrant Crypt Foci in Colon by Methylene Blue Staining

Aberrant crypt foci (ACF) represent preneoplastic lesions in colon carcinogenesis, characterized by abnormal tube-like glands in the colon and rectum, resulting from an increase in epithelial cell proliferation and resistance to apoptosis [35]. To assess the number and size of ACF, rat colons were expanded and fixed with 10% formaldehyde in PBS at pH 7.4. Subsequently, the colons were segmented into three sections, including the proximal, distal, and rectum segments. The flattened colons were then stained with 2% methylene blue. The size and the number of ACF were evaluated under a light microscope based on the criteria that followed Punvittayagul et al. [36]. 

### 2.5. Immunohistochemistry of PCNA in Liver and Colon Tissues

PCNA, a homotrimeric protein forming a ring structure encircling DNA, plays a crucial role in DNA replication during cell proliferation in the S-phase and can serve as a marker for cell proliferation [37]. The PCNA immunohistochemistry staining was followed by Chariyakornkul et al. [38]. After deparaffinization and rehydration processes, heat-induced target retrieval of the liver and the colon sections was performed by pressure cooking in citrate buffer (pH 6.0). Then, the endogenous peroxidase activity of tissue sections was blocked with 3% H_2_O_2_ for 15 min. Next, 1% skim milk was applied for 20 min at room temperature. After that, the tissue sections were then incubated with the mouse monoclonal anti-rat PCNA antibody (diluted at 1:1000 for liver tissues and 1:500 for colon tissues) at 4 °C overnight. After that, the slides were incubated with biotinylated antibodies and followed by using an Elite avidin–biotin complex kit. Visualization was achieved using DAB. Finally, the sections were counterstained with hematoxylin. The number of PCNA-positive cells was counted under a light microscope.

### 2.6. Determination of Apoptosis by Terminal Deoxynucleotidyl Transferase dUTP Nick-End Labeling (TUNEL) Assay in Liver and Colon Tissues

The TUNEL assay is a technique to investigate cell apoptosis by detecting DNA fragmentation through the addition of dUTPs to the 3′ hydroxyl terminus ends [34]. The TUNEL method was employed to assess cell apoptosis in the colon and the liver, following the instructions provided by the kit. The TUNEL-positive cells in the liver were determined under a light microscope. The number of TUNEL-positive cells was counted as apoptotic cells per liver area (mm^2^), while in the colon, TUNEL-positive cells were reported as the percentage of TUNEL-positive cells per crypt. 

### 2.7. Determination of Apoptotic Gene Expression by Quantitative Reverse Transcription Polymerase Chain Reaction (qRT-PCR) in the Liver and Colon Tissues

The qRT-PCR was used to determine the apoptotic gene expression following Punvittayagul et al. [39]. The apoptotic genes, including Bcl-2-associated X protein (*BAX*) and caspase-3 (*CASP3*), were analyzed. Initially, mRNA was isolated from frozen tissue of either the liver or the colon using PureZOL^TM^. Next, the first-strand cDNA was synthesized with a high-capacity cDNA reverse transcription kit. The cDNA was then subjected to amplification using the SYBR Green Master Mix Kit in a QuantStudio^TM^ 6 Flex real-time PCR detection system (QuantStudio^TM^6, Thermo Fisher Scientific, Waltham, MA, USA) using primers presented in Table 1. The expression levels of *BAX* and *CASP3* were normalized to the reference gene *ACTB* encoding β-actin and relatively calculated by the 2^−ΔΔCt^ method.

### 2.8. Statistical Analysis

The values were presented as mean ± standard deviation (S.D.). To ascertain the significant differences among groups in each experiment, one-way analysis of variance (ANOVA) was conducted, followed by a least-significant difference (LSD) test as a post hoc test. A *p*-value less than 0.05 was considered a significant difference.

## 3. Results

### 3.1. Effects of GTL, GTLH, and TPH on Rat Body Weight and Diet and Water Consumption

General observational data of the animals are presented in Table 2 and Table 3. All groups showed insignificant differences among their body weights, relative organ weights, including liver, kidneys, and spleen, and amounts of diet and water intake. Interestingly, TPH-treated carcinogen-receiving groups showed a significant increase in the amount of water intake (*p* = 0.037). Moreover, carcinogen-induced groups that received GTL, GTLH, and TPH had increased kidney weight when compared to rats that received carcinogens alone (*p* = 0.018, *p* = 0.026, and *p* = 0.030, respectively). In addition, the activities of alanine aminotransferase for the liver function test were not significantly observed in all groups of animals.

### 3.2. Effects of GTL, GTLH, and TPH on Preneoplastic Lesions in Liver and Colon of Rats

**Figure 3:**
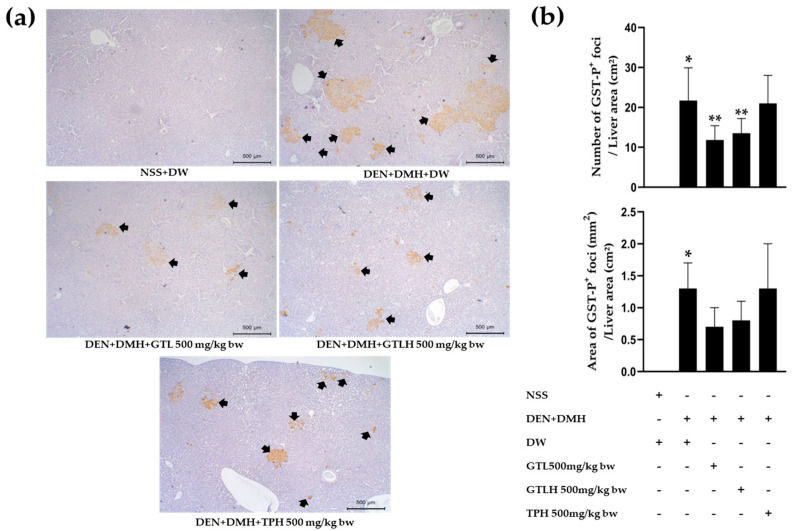
Effects of GTL, GTLH, and TPH on preneoplastic lesions (GST-P-positive foci) in liver tissues of carcinogen-induced rats. (**a**) Immunohistochemistry of GST-P-positive foci in liver tissue (40× magnification) treated with vehicle and samples (the black arrow is GST-P-positive foci). (**b**) The number and area of GST-P-positive foci in liver tissues. Values are expressed as mean ± S.D. NSS: normal saline solution; DW: distilled water; DEN: diethylnitrosamine; DMH: 1,2-dimethylhydrazine; GTL: glutelin; GTLH: glutelin hydrolysate; TPH: total protein hydrolysate. * and ** indicate significant differences at *p* < 0.05 when compared with NSS+DW group and with DEN+DMH+DW group, respectively.

The effects of GTL, GTLH, and TPH on preneoplastic lesions in the liver are shown in Figure 3. The NSS-treated rats did not show any GST-P-positive foci in all liver areas. None of the treatment samples induced preneoplastic lesions in the liver of NSS-treated rats. However, the liver of the carcinogen-induced rats presented a significant increase in GST-P-positive foci number and area compared to NSS-treated rats (*p* < 0.001). Interestingly, treatments of the carcinogen-induced rats with either GTL or GTLH statistically decreased the number of GST-P-positive foci (*p* = 0.002 and 0.009, respectively), while they did not affect the area of GST-P-positive foci. On the other hand, treatments with either TPH to carcinogen-induced rats did not decrease either the number or the area of GST-P-positive foci compared to carcinogen-induced rats.

**Figure 4:**
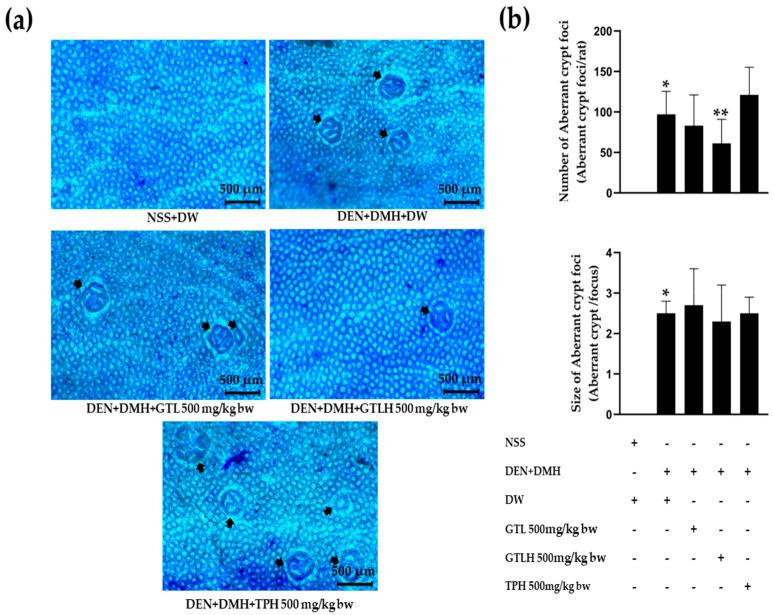
Effects of GTL, GTLH, and TPH treatments on ACF in colon tissues of carcinogen-induced rats. (**a**) The methylene blue staining of ACF (40× magnification) in the colon of a rat treated with DEN and DMH (Black arrow; ACF). (**b**) Number and size of ACF in rats. Values are expressed as mean ± S.D. NSS: normal saline solution; DW: distilled water; DEN: diethylnitrosamine; DMH: 1,2-dimethylhydrazine; GTL: glutelin; GTLH: glutelin hydrolysate; TPH: total protein hydrolysate. * and ** indicate significant differences at *p* < 0.05 when compared with the NSS+DW group and with the DEN+DMH+DW group, respectively.

Moreover, the effects of GTL, GTLH, and TPH on preneoplastic lesions in the colon are shown in Figure 4a,b. Carcinogen-induced rats presented a significant increase in ACF numbers and size when compared to NSS-treated rats (*p* < 0.001). Likewise, treatment of GTLH in carcinogen-induced rats significantly reduced ACF number when compared to rats receiving carcinogens alone (*p* = 0.029). Nevertheless, none of the sample-treated rats exhibited a decrease in the size of the ACF when compared to rats induced with carcinogens alone. Based on the results, we found that GTL and/or GTLH reduced the number of preneoplastic lesions in the liver and colon of carcinogen-induced rats. Riceberry GTL and GTLH may serve as cancer chemopreventive agents due to their ability to diminish preneoplastic lesions in the liver and colon of carcinogen-induced rats. Therefore, we further examined the underlying mechanisms related to cell proliferation and apoptosis using immunohistochemistry.

### 3.3. Effects of GTL, GTLH, and TPH on Cell Proliferation in the Liver and the Colon of Rats

**Figure 5:**
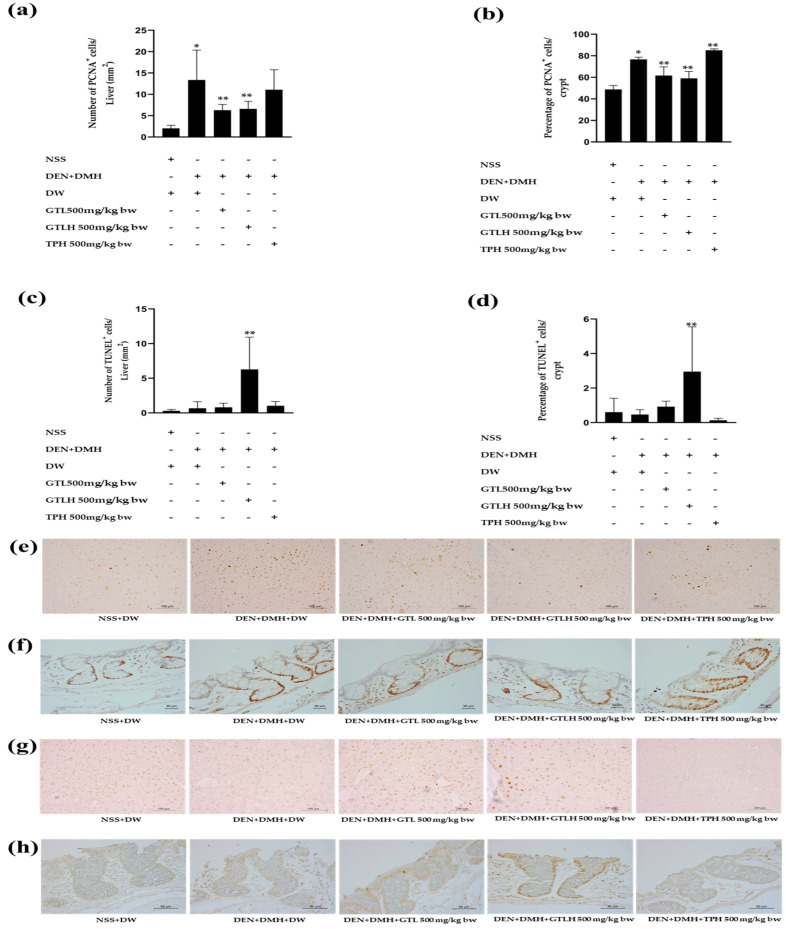
Effects of GTL, GTLH, and TPH on PCNA- and TUNEL-positive cells in the liver and colon tissues of carcinogen-induced rats. The number of PCNA-positive cells in the liver tissue (**a**). The number of PCNA-positive cells in the colon tissue (**b**). The number of TUNEL-positive cells in the liver tissue. (**d**) The number of TUNEL-positive cells in the colon tissue (**c**). The immunohistochemistry of PCNA-positive cells in the liver (**e**) and colon (**f**) tissues. The immunohistochemistry of TUNEL-positive cells in the liver (**g**) and colon (**h**) tissues. Values are expressed as mean ± S.D. NSS: normal saline solution; DW: distilled water; DEN: diethylnitrosamine; DMH: 1,2-dimethylhydrazine; GTL: glutelin; GTLH: glutelin hydrolysate; TPH: total protein hydrolysate. * and ** indicate significant differences at *p* < 0.05 when compared with the NSS+DW group and with the DEN+DMH+DW group, respectively.

The effects of GTL, GTLH, and TPH on cell proliferation in the liver and the colon are shown in Figure 5a,b. In the liver and colon, the number of PCNA-positive cells was significantly increased in the carcinogen-induced group compared to the NSS-treated rats (*p* = 0.01 and <0.001, respectively). The treatments with GTL and GTLH significantly reduced the number of PCNA-positive cells (*p* = 0.016 and 0.027, respectively), indicating a decrease in cell proliferation in both the liver and colon tissues compared to the carcinogen-induced rats. However, TPH treatments significantly increase the number of PCNA-positive cells in the colon of carcinogen-induced rats.

### 3.4. Effects of GTL, GTLH, and TPH on Apoptosis in the Liver and the Colon of Rats

The effects of GTL, GTLH, and TPH on apoptosis in the liver and the colon are shown in Figure 5c,d. In both the liver and colon, no significant differences in the number of TUNEL-positive cells were observed between the NSS-treated rats and the carcinogen-induced rats. Interestingly, treatment with GTLH in carcinogen-induced rats significantly increased the number of TUNEL-positive cells, indicating an enhancement of apoptotic cell death in both liver and colon tissues (*p* < 0.001 and *p* = 0.001, respectively).

### 3.5. Effect of GTLH on Expressions of Apoptotic Genes in the Livers and Colons of Rats

**Figure 6:**
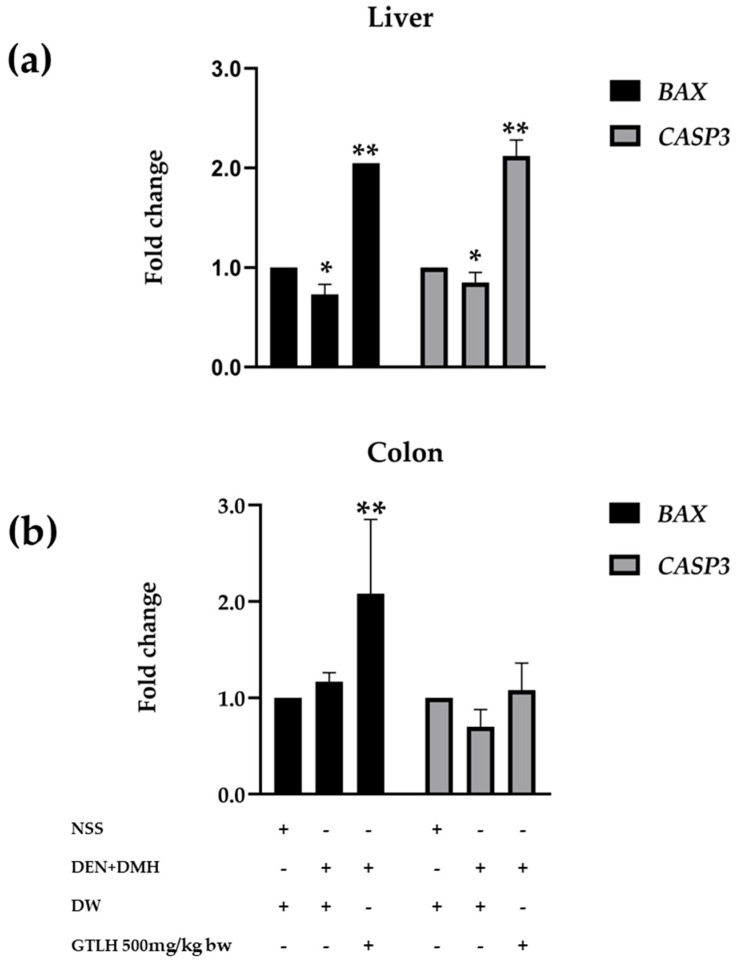
Effect of GTLH on the expressions of apoptosis genes in (**a**) liver tissue and (**b**) colon tissue. Values were expressed as mean ± S.D. NSS: normal saline solution; DW: distilled water; DEN: diethylnitrosamine; DMH: 1,2-dimethylhydrazine; GTLH: glutelin hydrolysate; *BAX*: BCL2-associated X; *CASP3*: caspase 3. * and ** indicate significant differences at *p* < 0.05 when compared with the NSS+DW group and with the DEN+DMH+DW group, respectively.

GTLH significantly promoted the expression of *BAX* in both liver and colon tissues (*p* < 0.001 and *p* = 0.047, respectively), while the effect of GTLH on the promotion of *CASP3* expression was only observed in the liver (*p* = 0.014) (Figure 6). There was no significant difference in *BAX* expression observed between the NSS-treated rats and the carcinogen-induced rats in both tissues. However, the *CASP3* expression in the colon presented no significant difference in all groups.

## 4. Discussion

Rice bran is composed of four proteins, including water-soluble albumin, salt-soluble globulin, alcohol-soluble prolamin, and alkaline-soluble glutelin [40]. Glutelin is a predominant protein, constituting about 22–40% of rice bran protein [41]. The limitations of glutelin as a pharmaceutical and in the food industry are the solubility and bioavailability of glutelin, attributed to its many disulfide bonds and hydrophobic interactions, underscoring its constraints [42]. Conversely, protein hydrolysates address these challenges by providing increased digestibility, greater bioactivity, superior functional characteristics, and broader use in specialized nutrition [29]. Various methods are available for protein hydrolysis, including chemical treatment, microbial fermentation, and enzymatic hydrolysis [43]. Among these techniques, enzymatic hydrolysis is one of the most commonly employed methods for producing bioactive hydrolysates [44]. This process typically involves the use of various enzymes, including Alcalase^®^, Flavourzyme^®^, papain, pepsin, trypsin, α-chymotrypsin, and pancreatin [45]. Enzyme-derived protein hydrolysates exhibit diverse biological activities, including antioxidant, antihypertensive, anti-inflammatory, and anticancer properties, making them valuable for health-promoting applications [21]. In line with our previous research, hydrolysates derived from Riceberry bran glutelin using Alcalase^®^ and Flavourzyme^®^ demonstrated significant antioxidant and antimutagenic effects in vitro [31]. Over the past decade, research on the cancer chemopreventive properties of Riceberry bran glutelin has remained inconclusive. In addition, protein extraction methods play a key role in the development of industrial biotechnology. Thus, two methods were examined, including protein-specific extraction and protein board extraction. The GTL extraction method, which involves an alkaline extraction followed by pH precipitation, is known for producing specific and high-purity protein [31]. Meanwhile, a non-precipitation alkaline method was used to extract total protein (TP), as it is recognized for its high yield, making it a promising option for industrial applications [32]. This work concerns how different protein extraction methods might influence anticancer activity. In this study, we aimed to assess the cancer chemopreventive effects of GTL, GTLH, and TPH in an animal model of carcinogen-induced liver and colon carcinogenesis.

Notably, general observational data, such as animal weight and diet intake of rats, did not present significant differences. However, the group of rats treated with TPH exerted a statistical change in water intake when compared to the carcinogen-induced rats. Previous studies have reported that TP preparations were contaminated with non-aggregated compounds such as sodium salt, sugar, fiber, minerals, Maillard reaction products, and modified peptides (lysinoalanine) [46,47]. Among these compounds, sodium salt has been reported as a thirst stimulator. The rats that received 0.9% (*w*/*w*) of sodium chloride solution significantly increased daily water ingestion [48]. Because dietary salt contributes to the osmotic burden of food, elevated plasma osmolality is known to trigger thirst [49]. In the TPH preparation method, the preparation of TP may result in salt contamination. Therefore, salt-contaminated TPH may contribute to increased water consumption behavior. Generally, relative organ weight is a critical parameter that provides insights into organ-specific toxicity, systemic effects, and the protective efficacy of potential agents [50]. Furthermore, we found that treatments with GTL, GTLH, and TPH significantly increase the relative kidney weight of rats compared to carcinogen-induced rats because of the slight reduction in body weight in these groups.

This study investigated the cancer chemopreventive potential of Riceberry GTL, GTLH, and TPH on the early stage of carcinogen-induced liver and colon carcinogenesis in rats. Preneoplastic lesions were evaluated by detecting GST-P-positive foci in the liver and ACF in the colon, both of which are used as biomarkers [35,51]. Carcinogen-induced rats presented a significant increase in the number of GST-P-positive foci in the liver. The results showed that both GTL and GTLH significantly reduced the number of GST-P-positive foci compared to the carcinogen-induced rat. We proposed that the cancer chemopreventive activity of GTL peptides may result from their hydrolysis by gastrointestinal enzymes, generating peptide fragments structurally similar to GTLH. The enzymes such as pepsin, trypsin, and chymotrypsin cleave peptide bonds at certain amino acid residues, particularly aromatic and hydrophobic side chains, similar to the cleavage sites targeted by Alcalase^®^ and Flavourzyme^®^ [52,53]. To support this explanation, an in vitro study on alkaline extraction of rice bran protein demonstrated that gastrointestinal digestion enhances its antioxidant and anti-inflammatory properties through the release of bioactive peptides [54,55]. These digested peptides can be absorbed through the small intestinal mucosa, enter the bloodstream, and be delivered to the liver [56]. However, the oral consumption of GTLH significantly decreased the number of ACF in colon tissue. Our previous study revealed that GTLH produced by enzymatic digestion contained four resistant peptides, including pyroglutamyl–valine, pyroglutamyl–aspartic acid, pyroglutamyl–asparagine, and pyroglutamyl–glutamine [31]. Moreover, these peptides could also be generated under microbial fermentation or high-temperature cyclization [57,58]. They exhibited in vitro antioxidant and antimutagenic activities and may contribute to carcinogenesis prevention in the liver and colon tissue [31]. We suggested that GTLH peptides may resist GI tract enzyme hydrolysis and contain these effective peptides rather than GTL. Then, resistant peptides from GTLH may be transported to the colon, promoting cancer chemoprevention on preneoplastic lesions in colonocytes. In particular, a study on pyroglutamyl peptides (pyroglu-Asn-Ile) derived from Japanese rice wine demonstrated their ability to reduce colitis-induced colonic dysbiosis in mice [58]. Previous studies have shown that Riceberry protein hydrolysates produced with Alcalase^®^ inhibit the proliferation of human colorectal adenocarcinoma HT-29 cell lines [30]. Kannan and colleagues found that small peptides (MW < 5 kDa) from Alcalase^®^ hydrolysis of rice bran proteins could induce cytotoxicity in human colorectal carcinoma cell lines (HCT-116) [59]. However, GTL and GTLH did not reduce the area or size of GST-P-positive foci and ACF. This observation suggested that their cancer preventive effects may not involve lesion size reduction but rather the inhibition of the transformation of normal cells into preneoplastic cells. Such effects could be mediated through mechanisms including the modulation of carcinogen biotransformation, prevention of DNA damage by chemical carcinogens or free radicals, promotion of DNA repair, and the induction of preneoplastic cell apoptosis [60]. Nonetheless, these bioactive peptides may be incapable of eliminating all cellular damage, allowing a subset of affected cells to progress toward a preneoplastic state. Moreover, we also observed the effect of TPH along with GLT and GTLH to confirm the specific source of bioactive peptide, not the whole protein. The alkali treatment of food proteins, commonly employed in food processing, leads to the creation of cross-linked amino acids like lysinoalanine [61]. Lysinoalanine residues have also been found to reduce digestibility and diminish nutritional quality, particularly in rodents. We suggested that the contamination of lysinoalanine may tend to promote preneoplastic progression in the colon tissue. These findings suggest that bioactive peptides derived from Riceberry bran GTL and GTLH exerted protective effects by inhibiting the early stage of carcinogenesis.

The balance between cell proliferation and apoptosis is critical in preventing cancer progression, particularly in the early stage of carcinogenesis. This study demonstrates that GTL and GTLH modulated these mechanisms by significantly suppressing cell proliferation in the liver and colon tissue. Consistent with other research, various bioactive peptides generated by commercial enzymes and gastrointestinal digestive proteases have been reported to inhibit cancer cell proliferation, such as the finding that rice bran peptide hydrolysate fractions with MW < 5 kDa presented a 50% inhibitory effect on cell proliferation of human hepatocellular carcinoma HepG2 cell lines [62]. In addition, the pentapeptide isolated from rice bran presented an inhibitory effect on cancer cell proliferation in human colorectal adenocarcinoma and carcinoma cell lines (Caco-2 and HCT-116) [63]. Moreover, previous studies reported that peptides derived from Riceberry bran hydrolyzed with Alcalase^®^ reduced the viability of a human colorectal adenocarcinoma cell line, namely HT-29, and a human colorectal carcinoma cell line, namely SW-620, by inducing apoptosis, cell cycle arrest, and senescence [30]. Furthermore, GTLH, which contains various pyroglutamyl peptides, was shown to induce apoptosis in liver and colon tissues, underscoring its potential in cancer chemoprevention. Likewise, cyclo-(Pro-Tyr), a compound isolated from the sponge *Callyspongia fistularis*, exhibited cytotoxic and apoptosis-inducing effects against the human hepatocellular carcinoma HepG2 cell line [64]. Moreover, cyclic peptides derived from *Laminaria japonica*, cyclo-(Trp-Leu-His-Val), exerted anti-colorectal carcinoma effects by inducing apoptosis in human colorectal adenocarcinoma Caco-2 cell lines and tumor-bearing mice [60]. We proposed that GTL peptides, digested by gastrointestinal proteases, exerted chemopreventive effects by inhibiting cell proliferation in the early stages of liver and colon carcinogenesis in rats. Furthermore, GTLH inhibited cell proliferation and induced apoptosis in the early stage of liver and colon carcinogenesis in rats. On the other hand, TPH may promote the development of preneoplastic lesions in the early stage of colon carcinogenesis in rats by inducing cell proliferation. It has been reported that high salt consumption could increase the risk of colorectal diseases such as inflammatory bowel disease, which was exacerbated by high salt consumption through the disruption of the immune–microbiota balance, resulting in a decrease in butyric acid production, a reduction of the Firmicutes-to-Bacteroidetes ratio, and a drop in Lactobacillus populations [65]. As discussed above, we suspected that salt contamination during TP and TPH preparation might interfere with their cancer chemopreventive properties. It is well known that salt exerts a strong influence on biochemical activities, leading to false interpretation of the results. To ascertain that salt contamination during TP preparation exerts an influence on the cancer chemopreventive properties, another set of experiments must be carried out by removing salt contamination with dialysis prior to cancer chemopreventive property tests in comparison with the TP without dialysis.

Interestingly, cancer cells can maintain cell survival, growth, and proliferation by apoptosis signaling suppression [66]. Therefore, many anticancer peptides promote cancer cell apoptosis and could be applied as cancer therapeutic agents. One of the apoptosis-induced pathways of anticancer peptides is involved with the mitochondrial outer membrane permeability (MOMP). These events are activated by the BCL-2 family pro-apoptotic proteins, including Bax and Bak [67]. The permeabilization releases intermembrane proteins like cytochrome c. This protein can bind with Apaf-1 and pro-caspase-9 to promote the protease cascade and apoptosis [68]. However, the molecular mechanisms underlying the inhibitory effects of GTLH remain unclear. Our findings indicated that GTLH significantly upregulated *BAX* and *CASP3* gene expression. In the colon, GTLH also markedly promoted *BAX* upregulation. According to a study on Cyclo(L-Leu-L-Pro) isolated from the sponge *Callyspongia fistularis*, the compound exhibited cytotoxic and apoptosis-inducing effects against human hepatocellular carcinoma HepG2 cells by significantly increasing the *BAX*/*Bcl-2* protein expression ratio and activating caspase-3 [64]. Moreover, peptides from brown seaweed, *Laminaria japonica*, exerted anticancer effects on human hepatocellular carcinoma HepG2 cell lines and triggered apoptosis via the caspase-dependent pathway, and caused G0/G1 cell cycle arrest [12]. Riceberry protein hydrolysate also induced apoptosis and G1/S cell cycle arrest in human colorectal carcinoma SW-620 and human adenocarcinoma HT-29 cell lines [30]. Especially, the study of the anti-hepatocellular carcinoma effect of sacha inchi peptides (SPs) or Inca peanut (*Plukenetia volubilis* L.) in human hepatocellular carcinoma HepG2 cell lines presented mitochondrial outer membrane permeabilization (MOMP) via the activation of a pro-apoptotic protein, which is Bax. This event allows the release of intermembrane proteins like cytochrome c, which can interact with Apaf-1 and pro-caspase-9 to initiate a protease cascade and ultimately apoptosis. They suggested that SPs inhibited proliferation and induced apoptosis by activating the mitochondrial pathway and caspase cascade [69]. Moreover, Ren and colleagues found that the human microbial preventive peptides (LL-37) treatment of human colon cancer cell lines led to the nuclear translocation of apoptosis-inducing factor (AIF) and endonuclease G (EndoG), key mediators of caspase-independent apoptosis [70]. This is consistent with previous studies on the cancer preventive properties of cyclic, pyroglutamyl, and other small peptides, which exhibited high specificity toward cancer cells, strong cell-penetrating ability, and apoptosis induction [71]. Based on our findings, we suggested that GTLH promoted apoptosis in liver cancer cells through the induction of *BAX* and *CASP3* gene expression, while in colon cells, apoptosis was induced primarily via *BAX* upregulation in a caspase-independent apoptotic pathway.

In addition, the concentration used in animal experiments should be based on the preliminary data of toxicity and pharmacological studies; however, we chose only one dose at 500 mg/kg b.w. based on the fact that all lysates had no toxicity to human PBMC at 400 μg/mL within 24 h, as in our previous report [31]. In addition, these peptides were derived from rice bran residues, which have less toxicity. A high single-dose design was then selected to adhere to the reduction principle of the Three Rs in animal experiments, while remaining scientifically sound [72]. Therefore, a high dose of 500 mg/kg b.w. was selected for this study, consistent with our regular protocols for testing other extracts from dietary sources. In the experiment, the rats received the lysates every week for five consecutive days, over a period of 10 weeks starting from day 1. In addition, they were injected with DEN on days 7, 11, and 18, and with DMH on days 7 and 14, to sufficiently induce hepatocarcinoma and colorectal cancers [73]. This experimental model was designed to observe events occurring at the very beginning of cancer development and to determine whether the tested compound can significantly reduce key markers, glutathione S-transferase placental form (GST-P)-positive foci in the liver, and aberrant crypt foci (ACF) formation in the colon. If so, it is suggested that the tested compound exerts chemopreventive properties.

The limitations of and further suggestions from these findings are listed for the short-term carcinogenesis model. It could be further investigated in the long term or for different cancer stages. Moreover, the exact mechanism of chemoprevention must be clearly elucidated. We also lack bioavailability data of these peptides, which should be observed before these peptides are further produced on a large scale.

## 5. Conclusions

GTLH derived from Riceberry bran residues exhibited cancer chemopreventive effects at the early stage of carcinogenesis in experimental rats induced by DEN and DMH for both liver and colon cancers. GTLH showed no adverse effects in normal rats at a dose of 500 mg/kg body weight and significantly reduced the number of preneoplastic lesions—GST-P-positive foci in the liver and ACF in the colon. GTLH inhibited cell proliferation by decreasing the expression of PCNA-positive cells. Moreover, GTLH promoted DNA fragmentation and cell apoptosis, as observed by the TUNEL assay. RT-PCR analysis confirmed that GTLH significantly upregulated the expression of *BAX* and *CASP3* in the liver and induced *BAX* in the colon. These findings suggest the potential of GTLH as a cancer chemopreventive agent. However, further research is necessary before its application as a functional food ingredient.

## Figures and Tables

**Table 1:** Primer sequence of target genes [39].

Target Gene	Primer Sequence	Primer Length
*BAX*	Forward-5′-TTCATC CAGGATCGAGCAGA-3′	21
	Reverse-5′-GCAAAGTAGAAGGCAACG-3′	18
*CASP 3*	Forward-5′-GGACCCGTCAATTTGAAAAA-3′	21
	Reverse-5′-CACCACGACTCCTACTGTAC-3′	20
*Β* *-* *Actin*	Forward-5′-ACAGGATGCAGAAGGAGATTAC-3′	22
	Reverse-5′-ACAGTGAGGCCAGGATAGA-3′	19

**Table 2:** Effect of GTL, GTLH, and TPH on body weight and amounts of water and diet intakes in rats.

Groups	Treatment	Body Weight (g)	Water Intake (mL/rat/day)	Diet Intake (g/rat/day)
1	NSS+DW	410.0 ± 31.8	26.8 ± 2.6	21.6 ± 3.0
2	NSS+GTL 500 mg/kg bw	441.0 ± 8.9	28.5 ± 4.1	22.0 ± 1.1
3	NSS+GTLH 500 mg/kg bw	417.0 ± 5.7	25.8 ± 2.8	20.4 ± 1.7
4	NSS+TPH 500 mg/kg bw	422.0 ± 25.1	29.8 ± 2.5	20.3 ± 1.6
5	DEN+DMH+DW	418.5 ± 33.0	25.7 ± 4.1	21.1 ± 1.8
6	DEN+DMH+GTL 500 mg/kg bw	385.0 ± 32.6	27.4 ± 5.3	22.9 ± 2.7
7	DEN+DMH+GTLH 500 mg/kg bw	391.9 ± 44.1	28.5 ± 3.5	23.9 ± 2.3
8	DEN+DMH+TPH 500 mg/kg bw	385.6 ± 34.4	31.0 ± 4.5 **	21.6 ± 0.8

Values are expressed as mean ± S.D. NSS: normal saline solution; DW: distilled water; DEN: diethylnitrosamine; DMH: 1,2-dimethylhydrazine; GTL: glutelin; GTLH: glutelin hydrolysate; TPH: total protein hydrolysate. ** indicates significant difference at *p* < 0.05, when compared with DEN+DMH+DW groups.

**Table 3:** Effects of GTL, GTLH, and TPH on relative organ weights in rats.

Groups	Treatment	Relative Organ Weight		
		Liver	Kidneys	Spleen
1	NSS+DW	3.56 ± 0.53	0.66 ± 0.10	0.16 ± 0.02
2	NSS+GTL 500 mg/kg bw	3.47 ± 0.47	0.59 ± 0.05	0.16 ± 0.02
3	NSS+GTLH 500 mg/kg bw	3.72 ± 0.43	0.65 ± 0.07	0.18 ± 0.02
4	NSS+TPH 500 mg/kg bw	3.55 ± 0.29	0.60 ± 0.05	0.16 ± 0.01
5	DEN+DMH+DW	3.94 ± 0.17	0.64 ± 0.04	0.20 ± 0.03
6	DEN+DMH+GTL 500 mg/kg bw	4.29 ± 0.54	0.74 ± 0.15 **	0.19 ± 0.03
7	DEN+DMH+GTLH 500 mg/kg bw	4.05 ± 0.41	0.73 ± 0.11 **	0.19 ± 0.04
8	DEN+DMH+TPH 500 mg/kg bw	4.15 ± 0.52	0.73 ± 0.10 **	0.19 ± 0.04

Values are expressed as mean ± S.D. NSS: normal saline solution; DW: distilled water; DEN: diethylnitrosamine; DMH: 1,2-dimethylhydrazine; GTL: glutelin; GTLH: glutelin hydrolysate; TPH: total protein hydrolysate. ** indicates significant difference at *p* < 0.05, when compared with DEN+DMH+DW groups.

## Data Availability

Data will be made available on request.
